# Genetically Encoded Biosensor Engineering for Application in Directed Evolution

**DOI:** 10.4014/jmb.2304.04031

**Published:** 2023-07-14

**Authors:** Yin Mao, Chao Huang, Xuan Zhou, Runhua Han, Yu Deng, Shenghu Zhou

**Affiliations:** 1 National Engineering Research Center for Cereal Fermentation and Food Biomanufacturing, Jiangnan University, 1800 Lihu Road, Wuxi, Jiangsu 214122, P.R. China; 2 Jiangsu Provincial Research Center for Bioactive Product Processing Technology, Jiangnan University, 1800 Lihu Road, Wuxi, Jiangsu 214122, P.R. China; 3 McKetta Department of Chemical Engineering, The University of Texas at Austin, Austin, TX 78712, USA

**Keywords:** High-throughput screening, biosensor, directed evolution, mutagenesis, microbial cell factory

## Abstract

Although rational genetic engineering is nowadays the favored method for microbial strain improvement, building up mutant libraries based on directed evolution for improvement is still in many cases the better option. In this regard, the demand for precise and efficient screening methods for mutants with high performance has stimulated the development of biosensor-based high-throughput screening strategies. Genetically encoded biosensors provide powerful tools to couple the desired phenotype to a detectable signal, such as fluorescence and growth rate. Herein, we review recent advances in engineering several classes of biosensors and their applications in directed evolution. Furthermore, we compare and discuss the screening advantages and limitations of two-component biosensors, transcription-factor-based biosensors, and RNA-based biosensors. Engineering these biosensors has focused mainly on modifying the expression level or structure of the biosensor components to optimize the dynamic range, specificity, and detection range. Finally, the applications of biosensors in the evolution of proteins, metabolic pathways, and genome-scale metabolic networks are described. This review provides potential guidance in the design of biosensors and their applications in improving the bioproduction of microbial cell factories through directed evolution.

## Introduction

In past decades, many microbial cell factories have been developed by systemic and synthetic biology engineering strategies to achieve the various requirements of humans [1, 2]. Extensive rational and irrational design strategies have been established to construct effective microbial cell factories. Rational design strategies need clear genetic background, easy genetic engineering, and knowledge of the interactions among different parts. However, due to the high complexity of global metabolic networks and protein structures in living cells, rational design strategies often fail to achieve the optimum phenotype and lead to additional challenges, such as unexpected enzyme performance, increased accumulation of by-products, and disturbance in energy and substance circulation [1, 3]. Furthermore, many non-model microorganisms have significant economic value for chemical biosynthesis but lack the genetic background information and genetic engineering tools, hindering the utilization of rational design strategie s. In this regard, irrational design strategies, such as directed evolution, have attracted increasing attention as they do not need the knowledge of the genetic background information to engineer a broad range of target DNA regions from specific genes to the whole genome [4]. Moreover, analyzing the evolution information will, in turn, promote an understanding of the genetic regulation mechanisms for a more rational design.

Directed evolution usually involves two steps, genetic diversity generation and optimum mutant screening. After decades of development, hundreds to billions of mutants have been easily generated according to the different requirements [5]. The existing approaches to generate genetic diversity include mainly PCR-based mutations, chemical and physical mutagenesis, and novel in vivo continuous evolution strategies such as PACE, CPR, MAGE, and CRISPR-based mutations [1]. In this regard, the main challenge of directed evolution has been how to identify and screen or select the optimum mutants from large libraries. To do so, genetically encoded biosensors combined with high-throughput screening equipment, such as fluorescence-activated cell sorting (FACS) and droplet-based microfluidics, can achieve the goal of rapid library screening [6, 7]. Biosensors can detect the concentration of specific metabolites and proportionally express reporter proteins to generate a detectable signal, achieving the goal of high-throughput screening [8].

**Fig. 1:**
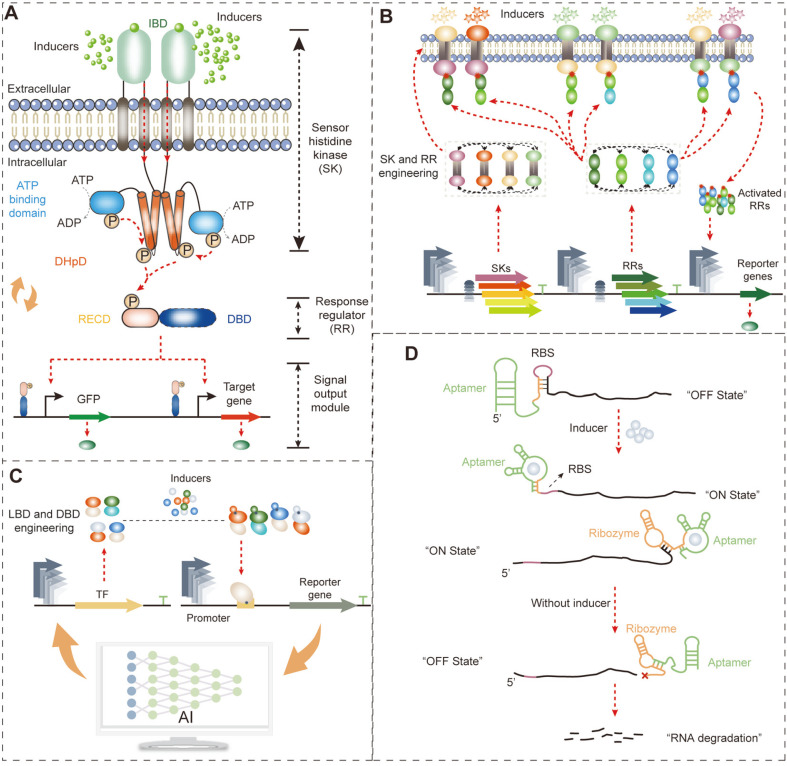
Mechanisms and engineering strategies of the genetically encoded biosensor. (**A**) The structure and signal transduction mechanism of TCBs. (**B**) Engineering strategies for changing the specificity and inducer dose curve of TCBs by domain swapping and expression level optimization. (**C**) Engineering strategies of TFBs. LBD or DBD engineering is often performed to change biosensor specificity, while promoter engineering is performed to regulate the expression level of TFs and reporter genes, which could manipulate the dynamic range, sensitivity, and leakage expression level of TFBs. (**D**) The mechanism of RBS-based riboswitch and ribozyme-based riboswitch.

Generally, genetically encoded biosensors include two-component biosensors (TCBs), transcription-factor-based biosensors (TFBs), and RNA-based biosensors (RNABs) (Fig. 1) [9]. TCBs contain a transmembrane sensor histidine kinase (SK) that detects the extracellular concentration of specific metabolites (Fig. 1A). TFBs and RNABs detect the intracellular concentration of specific metabolites. Hence, the application of these biosensors is determined by the spatial distribution differences of the detected metabolites [10]. Designing a satisfactory biosensor is challenging. The general biosensor engineering approaches have focused on regulating the expression level of biosensor components by promoter engineering, RBS engineering, and operator engineering to optimize biosensor performance [9]. Furthermore, computer-based protein engineering and artificial intelligence-based engineering approaches have also been raised in recent years. With the assistance of biosensors, the positive mutations in proteins, metabolic pathways, and whole-genome networks can be screened and enriched from a large library [11]. However, in the screening process, the biosensor detection range must cover the concentration of detected metabolites and generate a high enough dynamic range to distinguish background and positive mutants [12, 13]. Hence, biosensor designing is closely related to the requirements of directed evolution. This review discusses the mechanisms, engineering approaches, and applications of commonly used genetically-encoded biosensors in directed evolution. In particular, we compare the advantages and disadvantages of different biosensors in signal conduction and detection. Likewise, we describe approaches to protein, metabolic pathway, and genome scale-directed evolution and how they link with biosensor screening.

## Engineering Approaches of Genetically Encoded Biosensors 

In order to quantitatively monitor the performance of mutants from large mutation libraries, biosensors were developed in the past decades that could conduct a molecule concentration signal to a detectable signal, such as fluorescence and cell growth. Designing a biosensor with suitable performance, such as detection range, dynamic range, specificity, and leakage expression, is very important to satisfy the diverse requirements of directed evolution. Herein, we systemically discuss the recent advancements in biosensor engineering.

### TCB Engineering

TCB includes a sensor histidine kinase (SK), SK cognate cytoplasmic response regulator (RR), and an RR cognate promoter (Fig. 1A) [14, 15]. Specific inducers bind with the extracellular inducer binding domain (IBD) of SK to activate the SK by autophosphorylation [16]. The activated SK starts transforming the phosphate group from the dimerization and histidine phosphorylation domain (DHpD) of SK to the receiver domain (RECD) of RR, resulting in a conformational change in the DNA binding domain (DBD) to regulate the expression of the cognate promoter (Fig. 1A) [17]. Based on the above mechanism, TCBs detect extracellular concentration changes of small molecule compounds in directed evolution applications.

Currently, the focus of TCBs is mainly the engineering and design of the protein structure of SK and RR with the goal of modifying their recognition specificity (Fig. 1B) [4]. Theoretically, replacing the IBD of SK would shift the inducer specific of TCB because of their relatively independent and extracellular locations [18, 19]. In this regard, Ma *et al*. [20] and Luu *et al*. [21] replaced the IBD of NarX with the light-sensing domain of Cph1 from *Cyanobacteria Synechocystis* and the aromatic acid-sensing domain of PcaY from *Pseudomonas putida*, generating red light responsive and aromatic acid responsive TCBs, respectively. Furthermore, Soon Ho Hong and co-worker hybridized the methanol-sensing domain of MxaY from *Paracoccus denitrificans* or MxcQ from *Methylobacterium organophilum* XX with EnvZ from *E. coli*, obtaining the methanol-induced TCB MxaYZ-OmpR and MxcQZ-OmpR, respectively [22, 23]. However, no reliable swapping rules of IBD have been elucidated, hindering rational engineering of inducer recognition specificity. Lacking the precise 3D protein structure of the transmembrane SK is the main reason for the above shortage. We believe that with the development of biosensor engineering and protein structure resolution, a universally applicable IBD-swapping rule could be established in the future.

In addition to inducer recognition specificity, high specificity also exists between SK and RR (Fig. 1B). By computationally analyzing the interaction region of SK and RR, Skerker *et al*. identified the critical regions that significantly influence the recognition specificity of SK and RR, including seven conserved residues and the loop region of α1 to α2 in the histidine kinase domain (HKD) of SK [24]. In this regard, the authors established an MI + loop (mutual information + loop) design strategy to replace the conserved residues and loop region and change the specificity between SK and RR. This strategy has exhibited extensive reliability and applicability. The activated RR specifically recognizes the target DNA region to activate or repress expression of the cognate promoter (Fig. 1A). However, the natural cognate promoter often demonstrates leakage expression and undesired expression intensity, which is unsuitable for the screening in directed evolution. In this regard, replacing the DBD of RR probably could change the promoter recognition specificity of RR to obtain the desired TCB [25]. To do this, Schmidl *et al*. found the DBD of OmpR and NarL to be relatively independent and linked with RECD by a nonconservative linker surrounded by 39 conserved residues [26]. DBD swapping with the 39 conserved residues as the boundary achieved the goal of changing the DNA recognition specificity of RR. Finally, a TCB NarX-NarL_REC_-YdfI_DBD_ was constructed, achieving a 1,300-fold induction by NO_3_^-^. Overall, changing the recognition specificity of the inducer, RR, and promoter significantly expands the availability of TCBs for directed evolution.

The signal conduction of TCBs is based on the phosphorylation and dephosphorylation of SK and RR [15]. Hence, the interaction and expression levels between SK and RR directly influence the biosensor performance, such as detection threshold and dynamic range. In this regard, altering the phosphorylation and dephosphorylation states of SK can probably control the performance of TCB. As a proof of concept, Landry *et al*. established a Batchelor-Goulian mathematical model to quantitatively simulate the relationship between the detection threshold of TCB and the phosphatase activity of SK, and found that a proportional relation exists [27]. Analyzing the structural features of DHpD of SK, the authors found that a conserved phosphorylation sequence (GXGXG) existed in most SKs from different sources [27]. Engineering the second residue of GXGXG significantly regulates the detection threshold of TCB. Furthermore, regulating the relative expression level of SK and RR can also change the property of TCB. For instance, Yang *et al*. [28] used a panel of promoter-5´-UTR complexes with gradient strength to regulate the expression level and ratio of DcuS and DcuR. They found that the optimum expression ratio of DcuS:DcuR to be 46:54. In this scenario, the higher the expression level, the greater the dynamic range (Fig. 1B) [28]. However, why and how much the expression level of SK and RR could change the property of TCB remained unclear, which hindered the rational TCB designing. Mathematical modeling and artificial intelligence approaches were potential methods to overcome this challenge in the future to improve the design precision and efficiency. Collectively, engineering the SK and RR of TCBs regulates the detection threshold and dynamic range and changes the recognition specificities between the inducer and SK, SK and RR, and RR and promoter to obtain a satisfactory performance for serving the directed evolution.

### TFB Engineering

The transmembrane SK of TCBs allows them to detect only extracellular environmental changes. However, monitoring intracellular environments is also important in the process of directed evolution. Intracellular biosensors, including RNA-based biosensors (RNABs) and transcription-factor-based biosensors (TFBs), are often used to achieve this goal. Transcription factors (TFs) are repressor or activator proteins that regulate gene expression according to the changes in metabolite signals (Fig. 1C) [29]. TFs contain a functional ligand-binding domain (LBD) and DNA-binding domain (DBD) to recognize specific inducers and bind to promoter regions, respectively, to control gene expression (Fig. 1C). Using TFs to control the expression of reporter genes (typically fluorescent proteins or antibiotic-resistant genes) and constructing TFBs can achieve the goal of high-throughput detection of concentration changes of metabolites [30], such as amino acids [31, 32], succinic acid, naringenin, glucarate [33], 1-butanol [34], β-caprolactam [35], and putrescine [36]. In the high-throughput screening process of directed evolution, an appropriate TFB should be constructed with the desired performance, such as a wide detection range, low leakage expression, and high specificity and dynamic range.

Naturally existing TFs are abundant. For example, 304 candidate transcription factors were estimated to exist in *E. coli* [37]. They provide powerful tools for designing biosensors that can be employed in directed evolution. Recently, Li *et al*. identified a 3-dehydroshikimate sensing TF (*cusR*) from the genome of *E. coli* ATCC8739 by a method of selective comparative transcriptome analysis between the 3-dehydroshikimate producing and non-producing strains [38]. With the rapid development of bioinformatics, more and more natural TFs have been identified, and TF databases have been established, such as DBTBS (https://dbtbs.hgc.jp/), RegulonDB (https://regulondb.ccg.unam.mx/), RegTransBase (http://regtransbase.lbl.gov), and JASPAR (https://jaspar.genereg.net/). Using these databases, specific metabolite-inducible biosensors can be conveniently and efficiently constructed. However, the most interesting metabolites do not have the relative TFs in the above databases. To further broaden the recognition scope of TFBs, different LBDs and DBDs can be synthetically merged to create a single novel biosensor (Fig. 1C). In this manner, Chou *et al*. combined the DBD of AraC and GAL4 with isopentenyl diphosphate isomerase (*idi*), considered as the LBD, in *E. coli* and *S. cerevisiae*, respectively, resulting in two novel isoprenoid sensing TFs [39]. In doing this, the linker ligating the *idi* and DBD is significantly important for delivering the conformational change signal of IDI to the DBD to control gene expression. Furthermore, TF mutations can also be applied to alter the specificity to recognize analogs. In this regard, Snoek evolved the cis-muconic acid-inducible TF BenM by error-prone PCR to alter ligand specificity in response to adipic acid [19]. Likewise, the TF PcaV repressor, which was used for detecting hydroxyl-substituted benzoic acids, was further mutated to alter its ligand specificity towards vanillin and other closely related aromatic aldehydes, such as 3,4-dihydroxyaldehyde, 4-hydroxy-3-methylbenzaldehyde, and 2-hydroxybenzaldehyde, to generate the Van2 biosensor [40]. Overall, natural screening, LBD swapping, and TF mutations are effective approaches to obtaining the desired TFs for constructing TFBs.

The constructed TFs may suffer from inappropriate detection range and dynamic range due to inappropriate regulatory elements [41]. Generally, optimizing the strength of the promoter and RBS of TFs and reporters can fine-tune the performance of TFBs (Fig. 1C) [41, 42]. Traditional TFB optimization is used mainly in trial-and-error approaches, such as gradient changing the strength of promoter and RBS of TFs or/and reporters, to improve the sensitivity and dynamic range of OplR- [43], LasR- [44], and PadR- [45] based TFBs. Previous reviews have systemically concluded the trial-and-error optimization strategies of TFB [10, 41, 42]. Here, the focus is on the development of emerging artificial intelligence-based TFB optimization strategies (Fig. 1C). Ding *et al*. found that the RBS could significantly influence the dynamic range of TFBs by affecting the translation of TFs and reporter proteins [46]. In order to elucidate the rule between RBS and dynamic range, the authors designed an RBS library to fine-tune the translation of glucaric acid-sensing TF CdaR and sfGFP, and found that the medium strength of RBS generated a higher dynamic range. Then, the authors established a convolutional neural network-based deep learning platform (CLM-RDR) to precisely predict the TFB dynamic range with a given RBS sequence, obtaining a 72.2% accuracy rate [46]. Furthermore, regulating the transcription of TFs and reporter genes is also important for fine-tuning the dynamic range. Zhou *et al*. combined six promoters to regulate the expression of FapR and four different fapO positions, as well as 216 possible upstream enhancer sequences to regulate the expression of GFP. This generated large-scale genotype-phenotype association data for establishing a machine-learning prediction platform of the dynamic range [47]. Finally, the authors obtained an optimum FapR-fapO-based malonyl-CoA biosensor with the largest dynamic range (12.07-fold). Compared with the traditional trial-and-error approaches, this artificial intelligence strategy provides a time-saving and low-cost alternative for TFB optimization.

### RNAB Engineering

TFBs are the most widely used biosensors because they are stable and easily obtained. However, the transcription and translation processes of TFs and reporter proteins result in long response times, which is the main disadvantage of TFBs [10]. Comparatively, RNAB inducers respond at the mRNA level, significantly reducing the response time. RNABs include the RBS-based riboswitch and ribozyme-based riboswitch. The RBS-based riboswitch actives the translation of the reporter gene at the 5´-UTR region by exposing the RBS for ribosome access when the ligand is present (Fig. 1D). The ribozyme-based riboswitch controls the mRNA stability of the reporter gene at the 3´-UTR region by altering the ribozyme activity according to the concentration of the inducer (Fig. 1D) [48]. The aptamer is the region of inducer recognition and binding in RNAB. Binding of the inducer in the aptamer changes the secondary structure of RNAB through a transducer to expose RBS or split the 3´-UTR to control the expression of target genes. Hence, engineering the aptamer and transducer regions produces RNABs with the desired performance, such as specificity and dynamic range.

Recently, a systematic evolution of ligands by the exponential enrichment (SELEX) approach was established to screen a specific inducer-sensing aptamer from a large random sequence library in vitro [29]. In this process, the inducers are first immobilized on a solid matrix, and then the aptamer library is mixed with the affinity matrix. The nonfunctional RNAs are then washed off to enrich the functional aptamers [49]. In this manner, Jang *et al*. obtained a naringenin-responsive aptamer pool [50]. Coupled with the N10 random transducer screening, the authors obtained a naringenin-responsive RBS-based riboswitch with a 2.91-fold dynamic range [50, 51]. However, the aptamers screened from the random library in vitro often have no function in vivo. Naturally existing aptamers have conserved secondary and tertiary structures that are important for the functions of RBS exposure or ribozyme activity. Hence, engineering the inducer recognition region based on naturally existing aptamers to change the specificity, dynamic range, and sensitivity for obtaining the desired RNAB is a promising approach [52]. Porter *et al*. found that a three-way junction (3WJ) structure exists in natural aptamers, functioning to maintain its tertiary structure [52]. In order to efficiently construct functional aptamers in vivo, Porter *et al*. randomized the inducer binding region of *B. subtilis* *xpt-pbuX* guanine riboswitch, *V. cholerae* Vc2 cyclic di-GMP riboswitch, and *S. mansoni* hammerhead ribozyme in 3WJ. After screening, they successfully obtained 5-hydroxy-L-tryptophan (5HTP) and L-DOPA responsive aptamers [52]. Furthermore, the authors combined the screened aptamers with a fluorogenic aptamer (Broccoli) and a tRNA scaffold and constructed a stable and translation-free RNAB in *E. coli*, significantly reducing the biosensor response time [52]. Likewise, Pang *et al*. randomized the binding domain (18 nt) of the N-acetylneuraminic acid riboswitch, which comprises an aptamer and a hammerhead ribozyme, increasing the threshold, dynamic range, and sensitivity to 21.97 g/l, 3.25, and 45.9, respectively [53, 54].

Collectively, RNABs with the desired performance can be constructed by *de novo* aptamer and transducer screening or inducer binding region engineering approaches [55, 56]. Compared with TFBs and TCBs, RNABs offer shorter response times since the translation-free RNA is readily available for inducer binding and signal-to-output [41]. However, RNABs generally suffer from a low dynamic range, hindering the application of fluorescence proteins as reporters in directed evolution. TFBs and TCBs often can generate a dynamic range higher than 100 [9, 46]. Furthermore, TCBs functionally recognize extracellular inducers, while TFBs and RNABs recognize intracellular inducers. This determines the different high-throughput screening approaches in the directed evolution process [10]. Overall, the selection of different types of genetically encoded biosensors for application in directed evolution depends on the biosensor property and the spatial distribution scope of the inducer.

## Biosensor-Assisted Eirected Evolution

In the directed evolution process, high-throughput screening of desired phenotypes from large mutation libraries is the main bottleneck. Due to their high sensitivity and efficiency properties, genetically encoded biosensors have been increasingly applied to high-throughput screening [13]. In this process, biosensors detect mainly the accumulation of intra- or extra-cellular specific metabolites to indirectly reflect changes in genotype. In this regard, proteins, biosynthetic pathways of specific metabolites, and the whole genome of microorganisms can be evolved using genetically encoded biosensor-based directed evolution.

### Directed Evolution of Enzymes

**Fig. 2:**
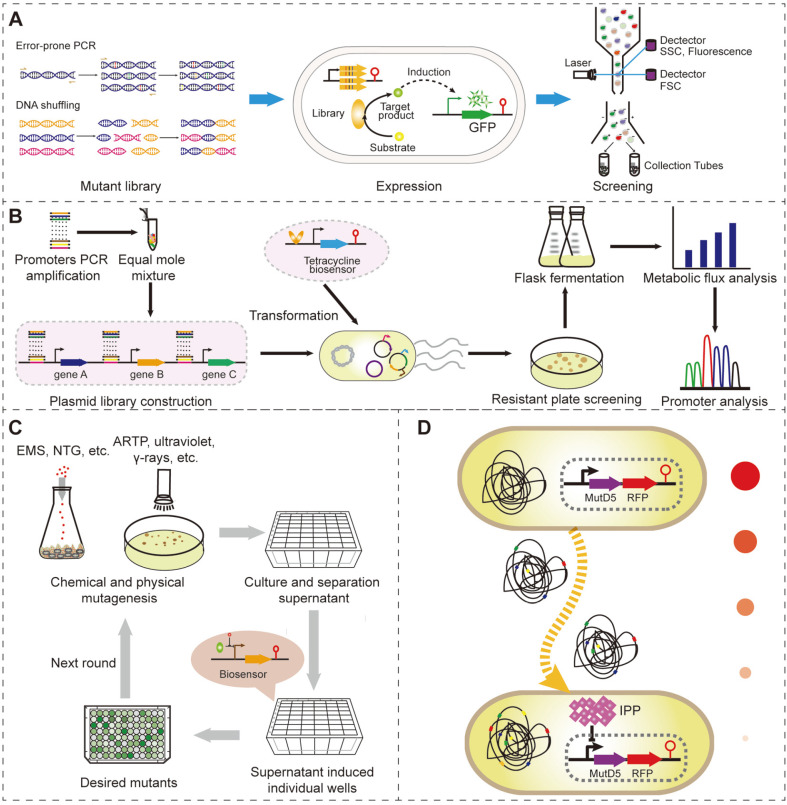
Genetically encoded biosensor-based directed evolution. (**A**) Directed evolution of enzymes with the assistance of FACS and genetically encoded biosensors. (**B**) Library construction and high-throughput screening to direct the evolution of the metabolic pathway with the assistance of biosensors. (**C**) Whole genome-directed evolution with the assistance of whole-cell biosensors in multi-well plates to avoid the accumulation of mutations in biosensors. (**D**) Continuous directed evolution with simultaneous mutation and selection in the cell growth process. Higher IPP production would generate lower expression levels of MutD5 and RFP (red fluorescence protein), resulting in the accumulation of positive mutations after screening the low red fluorescent cells.

The biosynthesis of any target product involves a series of enzymatic reactions [39]. An effective method for improving the accumulation of a target product is by enhancing enzyme activities [57]. Directed evolution has been widely used in protein engineering for enhancing enzyme properties [19, 40]. In doing so, enzymes are usually mutated by error-prone PCR, DNA shuffling, or DNA assembly PCR to generate a library for high-throughput screening by biosensors (Fig. 2A) [1, 4, 58]. High-activity enzyme mutants are catalyzed to generate more specific metabolites. Biosensors can transmit the concentration signal of metabolites into detectable fluorescence or cell growth signals for high-throughput screening [59]. Hence, superior mutants can be enriched and screened from large mutation libraries after several rounds of directed evolution.

Generally, RNABs are often used to control the expression of reporter genes, such as fluorescent protein genes or antibiotic-resistance genes, to sense and read out the concentration changes of specific metabolites that reflect the activity of the relative enzyme [48, 60]. For example, N-acetylneuraminate synthase (NeuB) is the rate-limiting step in the biosynthesis pathway of N-acetylneuraminic acid. In order to evolve NeuB, Yang constructed an N-acetylneuraminic acid-responding ribozyme-based RNAB by inserting an N-acetylneuraminic acid aptamer into the stem II of the hammerhead ribozyme [54]. The binding of N-acetylneuraminic acid promotes the self-cleavage of the ribozyme and reduces the stability of the mRNA of the reporter gene, thus controlling the expression level. Using this RNAB to control the expression of the tetracycline/H^+^ antiporter gene (*tetA*), the NeuB mutation library was screened in four rounds. A variant DN5/pB3 was obtained that produced 2.61 g/l N-acetylneuraminic acid, which was 23% higher than the wild-type enzyme [54]. In the screening process, the expression of *tetA* was closely related to cell growth under tetracycline and Ni^2+^ co-existing conditions. N-acylglucosamine 2-epimerase (*age_a_*) is another rate-limiting enzyme in the biosynthesis of N-acetylneuraminic acid. Using the same protein direct evolution method, Pang *et al*. mutated the *age_a_* by error-prone PCR and screened the library by the systemically optimized N-acetylneuraminic acid-responding ribozyme-based RNAB in three rounds. The best mutant DN5/pBac2B increased the titer by 54% (3.16 g/l) [53]. Comparatively, the application of TCBs in directed evolution is relatively fewer than the TBs and RNABs. A recent example was a pAPCE strategy developed by Morrison *et al*. through combining the phage-assisted continuous evolution (APCE) with CadC, a two-component sensor, to continuous evolution of protein-protein interactions [61]. In doing so, the periplasmic domain of CadC was replaced by a protein A and expressing a leucine zipper GCN4 ligated protein B. Wherein, leucine zipper GCN4 could promote the dimerization of protein B. If the dimerization protein B could interact and bind with protein A, the CadBA promoter would be triggered to express pIII, accumulating the positive mutants [62]. Using pAPCE, the affinity between disulfide-containing trastuzumab antibody and Her2-like peptide was improved two-fold [61].

FACS-based high-throughput screening can be performed when using fluorescent proteins as reporters in the directed evolution process. The sorting speed of FACS can reach 104~105 cells/s, significantly reducing screening time and labor costs (Fig. 2A) [1]. Due to the high fluorescence background of cells in FACS screening, a high expression level of fluorescent proteins generated by the activated biosensor is preferred and can increase the screening accuracy. Compared with RNABs, TFBs often exhibit a higher dynamic range and hence are more suitable for the application of FACS screening. RNABs are often used in growth-based screening by the expression of antibiotic-resistance genes as reporters in directed evolution. Using TFBs and FACS-based directed evolution, enzyme specificity and expression level can be easily manipulated. Using a LacI-based biosensor, Wu *et al*. directed the evolution of LacI and changed the specificity of the LacI variant (LacI-L5) to lactulose by FACS. Using the LacI-L5-constructed TFB, the authors developed a whole-cell lactulose biosensor to evolve cellobiose 2-epimerase, increasing the lactulose titer and cellobiose 2-epimerase expression level by 22- and 32-fold, respectively [63]. The mRNA structure change is speculated to be the reason for the expression improvement of the mutated cellobiose 2-epimerase. Except for the directed evolution of enzymes, improving the transfer efficiency of the transporter is also often considered in microbial cell factory engineering. As an example, Wang *et al*. established a xylose-responsive and XylR-based TFB in *S. cerevisiae*. Using this TFB, the authors evolved a hexose transporter (HXT14) through FACS screening and improved the xylose transportation efficiency to 6.5-fold [64].

Due to the co-existence of uninduced cells and dead cells in the screening library, a broad fluorescence histogram often arises in the FACS-based screening process, which significantly reduces the screening accuracy. Furthermore, the varied biosensor copy number in different cells also aggravates this challenge. To overcome this challenge, Michener *et al*. integrated a constitutive promoter-controlled RNAB into the genome, reducing the noise 2-fold. Using this screening platform, the authors evolved caffeine demethylase and improved the activity 33-fold [65]. Overall, biosensors used in the directed evolution of proteins are conducted by monitoring the specific small molecules that are catalyzed by enzymes or transferred by transporters [66]. FACS-based screening and growth-based screening are most often used in protein engineering.

### The Directed Evolution of Metabolic Pathways

The biosynthesis of microbial cell factories consistently requires maximizing the metabolic flux to the final products. Except for enzyme evolution to enhance activity, gene expression level optimization is another focus for pathway optimization [67]. Pathway optimization is often conducted by regulating each gene’s translation and/or transcription level in the target metabolic pathway. However, such a regulation strategy generates numerous combinations with gradient expression levels of genes, which is challenged in the construction and characterization of these combinations [68]. To overcome this challenge, researchers generally mix the gradient strength promoters or RBSs and ligate them with target genes to construct a library. Following biosensor-assisted high-throughput screening, the optimum combinations can be obtained, achieving the goal of metabolic pathway optimization (Fig. 2B).

In the pathway optimization process, directly optimizing the expression level of a rate-limiting enzyme is a feasible and efficient approach. Focusing on the optimization of the rate-limiting enzyme (AroD) of L-phe production, Liu *et al*. constructed a random sequence from the RBS library that controlled the expression of AroD. Pathway evolution was performed with the assistance of TyrR-based TFB and FACS-based screening. This resulted in 5.79 g/l L-phe being produced with an 80% improvement over the original strain [69]. Sometimes, optimizing only the expression of the rate-limiting enzyme cannot maximize the metabolic flux. Hence, simultaneously optimizing the expression of all enzymes in the synthetic pathway is important. To systemically optimize the synthetic pathway of N-acetylneuraminate, Yang *et al*. ligated gradient-strength RBS libraries upstream of GlcN-6-P synthase (glmS*), a GlcN-6-P N-acetyltransferase (*GNA1*), GlcNAc 2-epimerase (*age_a_*), and NeuAc synthase (*neuB_c_*). Using N-acetylneuraminate-responsive RNAB screening, the titer improved by 39%compared with the original strain [53, 54].

Furthermore, promoters or promoter-5´-UTR complexes (PUTRs) [67] are also functional in pathway optimization. Xu *et al*. constructed a glycolate-responsive TFB and developed a 48-well deep-well plate and agar plate high-throughput screening platform by using *gfp* and *tetA* as reporters, respectively (Fig. 2B) [70]. The constructed biosensor exhibited a wide detection range (0.1–200 mM) and a high dynamic range (79-fold), showing high reliability in screening accuracy (R^2^=0.903). Finally, the authors inserted 22 gradient PUTRs upstream of genes of the glycolate synthetic pathway (*gltA*, *aceA*, and *ycdW*), generating a library with 10,648 possible combinations. Screening this library generated an optimum strain that produced 40.9 g/l glycolate. Furthermore, a more complicated pathway library can also be optimized by biosensor-assisted directed evolution, such as simultaneously optimizing the gene sources and expression level of the naringenin synthetic pathway [71]. Overall, biosensor-assisted screening enables metabolic pathway optimization in a high-throughput manner and significantly reduces the workload. In this scenario, only the library size of the combinate metabolic pathway is the main bottleneck in pathway optimization. This arises from the high efficiency in the screening process and the limited ligation and transformation efficiency in the pathway construction process.

### Directed Evolution to Enhance the Performance of Microbial Cell Factories

The product synthesis pathways of organisms are connected in a complex network system. Multiple genes often control the specific phenotype of cells. Furthermore, the required precursor, energy, and co-factors are also supplied by the global metabolic network of microbial cell factories. Hence, engineering at the global-genome level to reconstruct the metabolic network has attracted increasing attention. It can lead to changes in many complex cell phenotypes controlled by multiple genes and ultimately obtain the desired excellent phenotype.

Mutagenesis, such as ultraviolet mutagenesis and atmospheric pressure room temperature plasma (ARTP) mutagenesis, is the most commonly used approach in global-genome perturbation (Fig. 2C) [72]. In the directed evolution process, biosensors are usually first transformed in the producing strains and then mutagenesis is conducted [38, 69, 73, 74]. The mutated library is then cultured and screened by FACS, multi-well plates, or agar plates. However, in the mutation process, biosensors exist in the cell and probably are also being mutated, thus generating strong fluorescence or antibiotic resistance and reducing the screening accuracy. To overcome this challenge, dual reporters are usually applied to reduce the probability of unexpected mutations on biosensors. For example, Qiu *et al*. used the kanamycin resistance gene and *gfp* as the reporter gene to provide a cross-validation for screening malonyl-CoA accumulation strains during cell growth and detecting fluorescence levels [75]. Directed evolution usually needs multiple rounds to accumulate beneficial mutations. With increased rounds of mutagenesis, mutations in biosensors simultaneously accumulate, thus possibly changing the performance of biosensors and increasing invalid isolates. In this regard, using biosensor-containing cells as whole-cell biosensors to screen mutants in multi-well plates (Fig. 2C) [76] or microfluidic device-based FACS screening [45] are effective solutions. In the multi-well plate screening process, microbial cell factories are first mutated, and single colonies are cultured in multi-well plates. The supernatants of the cultures are used to induce the expression of the whole-cell biosensor in a new multi-well plate to identify positive mutants [76]. Whole-cell biosensors and mutated single cells can also be embedded in micro-droplets by microfluidic devices and followed by FACS screening to efficiently screen positive mutants and avoid the mutations in biosensors [45].

Mutations, screening, and characterization are the three steps of directed evolution. A single round of directed evolution generates only limited positive mutants that can easily be missed in the screening process. Although multiple rounds of directed evolution can significantly accumulate beneficial mutants, time and labor are consumed. Hence, continuous directed evolution was developed to overcome the above challenges [1]. Continuous directed evolution can simultaneously conduct the mutation and screening steps to accumulate beneficial mutants in a given selected condition [77]. In doing so, mutagenesis is accompanied by genome DNA replication. The generally used mutagenesis approaches in continuous directed evolution include mainly fallible DNA polymerase-based mutagenesis [78, 79], MAGE [80], CRISPR-X [81, 82], and CREATE [83, 84]. According to the concentration changes of the detected compounds, biosensors are coupled to repress the growth of negative mutants or regulate the mutation rate of positive mutants. For example, Chou *et al*. established the feedback-regulated evolution of phenotype (FREP) system by using an isopentenyl diphosphate-responsive biosensor to control the expression of MutD5, an engineered fallible proofreading DNA polymerase III, and generate random mutations with cell replication (Fig. 2D) [79]. The expression of MutD5 would then increase the mutation rate in the genome. When a mutant produced higher isopentenyl diphosphate, the expression level of MutD5 was reduced by the feedback repression of the biosensor, thus accumulating the beneficial mutations (Fig. 2D). Finally, using FREP to evolve the production of lycopene in *E. coli*, the titer improved 6.8-fold after 432 h of continuous directed evolution [79]. Biosensor and MAGE combination also could achieve the goal of continuous directed evolution. Raman *et al*. systemically optimized the expression level of TF (TtgR and CdaR) and reporter (TolC), obtaining superior naringenin and glucaric acid responsive biosensors [85]. TolC is an outer membrane protein that could export toxic compounds, such as SDS and colicin E1, and generate resistance. Thus, in order to continuously directed evolute *E. coli* to enhance chemical production, Raman *et al*. using the MAGE method randomly mutated the target genes that were screened by FBA prediction, and screened positive mutants by biosensors that linked the product titer with cell tolerance of SDS and colicin E1, improving the titer of naringenin and glucaric acid by 36-and 22-fold, respectively, after four rounds of evolution [85]. In the evolution process, the high SDS or colicin E1 tolerance probably not mean a high titer since the cheater strains with low titer and high tolerance also have a high potential to arise and accumulate. Hence, using TolC as a reporter allows a positive selection (SDS) and negative selection (colicin E1), significantly reducing the accumulation rate of cheater strains [85, 86].

## Conclusions and Prospects

Collectively, genetically encoded biosensor-based directed evolution has enabled the rapid and efficient engineering of proteins, metabolic pathways, and global metabolic networks [9]. Establishing a genetically encoded biosensor with the desired performance, such as specificity, dynamic range, and detection range, is the primary requirement for the application in directed evolution. In this regard, promoters, RBS, and operator engineering approaches have been established to regulate the expression of biosensor components for performance optimization [41]. Furthermore, RNA and protein domain swapping can effectively change the specificity [13]. The constructed biosensors generally respond to the extracellular or intracellular concentration of single specific compounds by producing detectable signals such as fluorescence or cell growth. Hence, coupled with the desired biosensors and the different mutagenesis approaches, the expected genotype can be screened through high-throughput screening of the detectable phenotype. In doing so, TCBs are functional for detecting the extracellular concentration changes of chemicals, and are thus applicable in directed evolution for improving the production of extracellular products. TFBs and RNABs are functional in detecting intracellular chemicals [10]. The application of different types of biosensors should depend on the spatial distribution of the detected chemicals.

Currently, precisely and efficiently designing biosensors is the most significant challenge. The time and labor costs of trial-and-error approaches in obtaining the desired biosensors are mainly in the optimization of the expression level and structure of TFs, SKs, RRs, and riboswitches [41]. Deep learning and machine learning-based artificial intelligence technology are beginning to be introduced into the design of biosensors to overcome this challenge, significantly improving the design precision and efficiency [46, 47]. However, applying artificial intelligence in biosensor design is premature as it lacks the unified big data collection approaches for training artificial intelligence models. In this aspect, in light of the rapid development of omics technology and DNA-chip synthetic technology, we believe more efficient and precise big data will be mined for model training in the future. With the assistance of precisely designed biosensors, directed evolution can be efficiently conducted to generate optimum mutants.
